# Lung-Selective Delivery of mRNA-Encoding Anti-MERS-CoV Nanobody Exhibits Neutralizing Activity Both In Vitro and In Vivo

**DOI:** 10.3390/vaccines12121315

**Published:** 2024-11-24

**Authors:** Yuhang Zhang, Chongyu Tian, Xinyang Yu, Guocan Yu, Xuelian Han, Yuan Wang, Haisheng Zhou, Shuai Zhang, Min Li, Tiantian Yang, Yali Sun, Wanbo Tai, Qi Yin, Guangyu Zhao

**Affiliations:** 1 School of Basic Medical Sciences, Anhui Medical University, Hefei 230032, China; yuhang@stu.ahmu.edu.cn (Y.Z.); haisheng@ahmu.edu.cn (H.Z.); 2 State Key Laboratory of Pathogen and Biosecurity, Academy of Military Medical Sciences, Beijing 100071, China; hanxuelian@bmi.ac.cn (X.H.); wangyuan@bmi.ac.cn (Y.W.); limin@bmi.ac.cn (M.L.); ytt_tjutcm@163.com (T.Y.); 18852551076@163.com (Y.S.); 3 Institute of Infectious Diseases, Shenzhen Bay Laboratory, Shenzhen 518132, China; tiancy@sxau.edu.cn; 4 College of Veterinary Medicine, Shanxi Agricultural University, Jinzhong 030031, China; 5 Key Laboratory of Bioorganic Phosphorus Chemistry & Chemical Biology, Department of Chemistry, Tsinghua University, Beijing 100084, China; yuxinyang@tsinghua.edu.cn (X.Y.); guocanyu@mail.tsinghua.edu.cn (G.Y.); 6 Laboratory of Advanced Biotechnology, Academy of Military Medical Sciences, Beijing 100071, China; 7 School of Life Sciences, Anhui Agricultural University, Hefei 230036, China; shuaiz2024@stu.ahau.edu.cn; 8 Public Health School, Mudanjiang Medical University, Mudanjiang 157011, China

**Keywords:** mRNA, nanobody, lung-selective LNPs, MERS-CoV

## Abstract

**Background/Objectives**: The Middle East Respiratory Syndrome Coronavirus (MERS-CoV) is a highly pathogenic virus causing severe respiratory illness, with limited treatment options that are mostly supportive. The success of mRNA technology in COVID-19 vaccines has opened avenues for antibody development against MERS-CoV. mRNA-based antibodies, expressed in vivo, offer rapid adaptability to viral mutations while minimizing long-term side effects. This study aimed to develop a lung-targeted lipid nanoparticle (LNP) system for mRNA-encoding neutralizing nanobodies against MERS-CoV, proposing a novel therapeutic strategy. **Methods**: An mRNA-encoding nanobody NbMS10 (mRNA-NbMS10) was engineered for enhanced stability and reduced immunogenicity. This mRNA was encapsulated in lung-selective LNPs using microfluidics to form the LNP-mRNA-NbMS10 system. Efficacy was assessed through in vitro assays and in vivo mouse studies, focusing on antigen-binding, neutralization, and sustained nanobody expression in lung tissues. **Results**: The LNP-mRNA-NbMS10 system expressed the nanobody in vitro, showing strong antigen-binding and significant MERS-CoV pseudovirus neutralization. In vivo studies confirmed selective lung mRNA delivery, with high nanobody expression sustained for up to 24 h, confirming lung specificity and prolonged antiviral activity. **Conclusions**: Extensive in vitro and in vivo evaluations demonstrate the LNP-mRNA-NbMS10 system’s potential as a scalable, cost-effective, and adaptable alternative to current MERS-CoV therapies. This innovative platform offers a promising solution for preventing and treating respiratory infections, and countering emerging viral threats.

## 1. Introduction

Since the first outbreak of Middle East Respiratory Syndrome Coronavirus (MERS-CoV) in 2012, it has remained a major global public health concern [1,2,3]. The virus causes severe respiratory illness and has a high mortality rate, posing a persistent threat to global health [4,5]. Despite significant progress in understanding MERS-CoV epidemiology and transmission patterns, effective prevention and treatment measures are still lacking [6,7,8]. Global demand for vaccines and antiviral drugs continues to rise, but high costs, limited accessibility, and frequent dosing requirements of current treatments remain major barriers. Additionally, the rapid mutation of the virus and the emergence of immune escape variants further complicate the situation [9,10,11]. Therefore, there is an urgent need to develop new prevention and treatment strategies that address current strains, allow rapid resynthesis to match new variants, and are both economically viable and scalable for widespread global use.

The use of mRNA nanobodies offers several key advantages over conventional therapeutics [12]. First, the small size of nanobodies enables rapid tissue penetration and access to antigens that may be inaccessible to larger antibodies [13,14,15]. This is particularly advantageous in the respiratory tract, where traditional antibodies may have limited reach. Moreover, the modular nature of mRNA allows for quick modifications to the nanobody sequence in response to viral mutations, maintaining efficacy against evolving MERS-CoV strains [16,17]. This adaptability is critical in the dynamic landscape of infectious diseases, where new strains and variants can emerge unpredictably. Incorporating pseudouridine into the mRNA sequence further enhances the stability of the mRNA and reduces its immunogenicity, which is crucial for the in vivo longevity and effectiveness of the nanobodies [18]. This modification ensures that the mRNA nanobodies persist long enough to elicit a robust immune response while minimizing potential adverse reactions, providing a safer and more potent therapeutic option.

In addition, using lipid nanoparticles (LNPs) for encapsulating and delivering mRNA nanobodies offers a targeted approach, minimizing off-target effects and reducing the required dosage for therapeutic intervention. The LNPs can be tailored to preferentially accumulate in the lungs, the primary site of MERS-CoV infection, thereby increasing the local concentration of the nanobody and enhancing its protective effect [19,20]. This targeted delivery also has the potential to reduce the systemic side effects often seen with broad-spectrum antivirals. In summary, combining mRNA technology with nanobodies provides a highly specific, rapidly deployable, and cost-effective solution for combating MERS-CoV and other infectious diseases. This approach shows great potential for developing next-generation antiviral therapies.

In this study, we used LNPs to deliver mRNA encoding the potent nanobody NbMS10, which our team previously developed to target the receptor-binding domain (RBD) of the MERS-CoV spike protein [21]. Our system, termed LNP-mRNA-NbMS10, was designed to express a robust neutralizing antibody against MERS-CoV when administered to the pulmonary system. We conducted comprehensive in vitro and in vivo studies to evaluate the efficacy of this mRNA nanobody in neutralizing MERS-CoV at the cellular level and protecting mice from lethal infection. Our findings underscore the potential of this innovative mRNA-encoded nanobody approach as a promising alternative to current therapeutic interventions and preventive strategies against MERS-CoV.

## 2. Materials and Methods

### 2.1. Generation of Modified mRNA

The NbMS10 sequence was modified by adding a signal peptide to its N-terminus. The nucleotide sequences encoding both the signal peptide and NbMS10 were synthesized by the Beijing Norsee Genomics Research Center Co., Ltd. (Beijing, China). Additionally, a human Fc tag was attached to the C-terminus of NbMS10 using the plasmid pFUSE-hIgG1-Fc2. To improve mRNA stability and translation efficiency, NbMS10 was incorporated into a plasmid construct containing 3′ untranslated regions (3′UTR), a poly(A) tail, and 5′ untranslated regions (5′UTR).

The linearized plasmid served as a template for in vitro transcription using a commercial transcription (Vazyme, Nanjing, China, Cat. No. DD4202). During transcription, all UTPs were fully substituted with N1-methylpseudouridine, followed by capping (Vazyme, Cat. No. DD4110), and purification (Vazyme, Cat. No. N412).

### 2.2. Lipid Nanoparticle Preparation

The lung-selective lipid nanoparticle formulation was prepared according to the previous method [19]. Briefly, the ethanol-based mixture includes ionizable lipid, DOPE, cholesterol, PEGylated lipid, and a targeting lipid, with molar percentages of 24.5%, 4.7%, 20.0%, 0.8%, and 50%, respectively. The solution containing mRNA and the ethanolic mixture were quickly combined at a volume ratio of 3:1 (aqueous to ethanol) and a mass ratio of 40:1 (total lipids to mRNA).

The synthesis of ionizable lipid and targeting lipid was conducted as previously described [19]. Briefly, for ionizable lipid synthesis, oleic acid (100 mmol), thionyl chloride (150 mmol), and *N*,*N*-dimethylformamide were dissolved in toluene (150 mL). The mixture was heated to 60 °C for 4 h. Following this, both toluene and thionyl chloride were removed through vacuum distillation, yielding oleoyl chloride, which was utilized without undergoing additional purification steps. Oleoyl chloride (20 mmol) and triethanolamine (10 mmol) were dissolved in dichloromethane (DCM). Triethylamine (22 mmol) was then added dropwise while stirring at 0 °C. After the mixture was stirred overnight and filtered, the solution was diluted with DCM and washed with water. The organic phase was dried over Na_2_SO_4_ and subsequently evaporated to obtain the crude product. This crude product was further purified by flash column chromatography using a DCM/methanol mixture (9:1), yielding the ionizable lipid as a brown liquid.

For targeting lipid synthesis, ionizable lipid (1 mmol) and methyl iodide (2 mmol) were combined in 10 mL of acetonitrile. The mixture was then heated at 70 °C for 24 h. Subsequently, the solvent and excess methyl iodide were removed by evaporation, yielding the desired targeting lipid as the final product.

### 2.3. The In Vitro Expression of NbMS10

293T cells were seeded in 6-well plates under optimal growth conditions, and the culture medium was replaced with Opti-MEM (Gibco, Waltham, MA, USA, Cat. No. 31985070) 2 h before transfection. A total of 2.5 micrograms of LNP-mRNA-NbMS10 was transfected into the cells.

### 2.4. SDS-PAGE and Western Blot

At 48 h post-transfection, the supernatant was collected for analysis. The supernatant (40 μL) was mixed with an equal volume of 5× SDS loading buffer (Cowin Biotech, Taizhou, China, Cat. No. CW0027S) and subjected to thermal denaturation at 100 °C for 10 min. After brief centrifugation, the supernatant was loaded onto 12% Tris-glycine SDS-PAGE gels for electrophoresis.

Proteins were transferred onto nitrocellulose membranes and blocked overnight at 4 °C using PBST (phosphate-buffered saline with tween-20) containing 50 g/mL of skim milk powder. The HRP-conjugated goat anti-human IgG (H+L) tag antibody (Beyotime, Wuxi, China, Cat. No. A0201) was diluted 1:1000 and incubated at 37 °C for 2 h. The membranes were washed with TBST (Tris-Buffered Saline with Tween-20) for 5 min, repeated 4 times. Finally, the membrane was imaged using a GE Amersham Imager 600 (GE Healthcare, Chicago, IL, USA) after excitation with an ECL luminescent solution.

### 2.5. RNA Agarose Gel Electrophoresis

The 0.36 mg of agarose was dissolved in 30 mL of 1× TAE buffer. The mRNA sample was denatured by heating at 90 °C for 2 min, followed by immediate cooling on ice for 2 min. The entire sample was then loaded onto the gel. RNA electrophoresis was performed, and the results were visualized using a UV transilluminator.

### 2.6. Luciferase or eGFP mRNA Delivery

Female BALB/c mice, weighing 18–19 g, were used in this experiment. Each mouse received an intravenous injection of 19 μg of luciferase mRNA. At designated time points, the animals were given an intraperitoneal injection of D-luciferin (150 mg/kg). Bioluminescence imaging was conducted using the IVIS Lumina system (Perkin Elmer, Shelton, CT, USA). A similar procedure was followed for the intravenous delivery of eGFP mRNA. Post-treatment, the mice were sacrificed, and their lungs were harvested for confocal microscopy examination.

### 2.7. ELISA

NbMS10 levels in both sera and lung tissues were quantified using an enzyme-linked immunosorbent assay (ELISA). A 96-well plate was coated with the MERS-CoV S1 protein (Sino Biological, Beijing, China, Cat. No. 40069-V08H) to a final concentration of 0.25 μg/L and incubated overnight at 4 °C. The next day, the plate was blocked with 5% BSA in PBS at 37 °C for 1 h. After blocking, serum samples were added and incubated at 37 °C for 2 h. Next, an HRP-conjugated goat anti-human IgG (H+L) tag antibody (Beyotime, Cat. No. A0201), diluted 1:250, was added and incubated at 37 °C for 1 h. A standard curve was generated using a purified NbMS10 antibody for quantification.

### 2.8. Pseudovirus Neutralization Assay

A plasmid encoding the MERS-CoV S protein gene and an HIV luciferase vector was co-transfected into 293T cells using Invitrogen Lipofectamine 3000 (Thermo Fisher, Waltham, MA, USA, Cat. No. L3000015). The supernatant, collected 48 h post-transfection, contained pseudovirus particles, which were then stored at −80 °C. The pseudovirus was co-incubated with the samples for 1 h at 37 °C in a 5% CO_2_ incubator. After incubation, the samples were added to Huh-7 cells. Following a 48 h incubation, luminescence was measured using the Bright-Lite Luciferase Assay System (Vazyme, Cat. No. DD1204) and a chemiluminescence detector for analysis.

### 2.9. Statistical Analysis

All data were statistically analyzed using GraphPad Prism version 9.0 and OriginPro 2021 software. Unless otherwise noted, data from all experiments are presented as the mean ± standard error of the mean (SEM). Statistical significance was determined by one-way analysis of variance (ANOVA) with post hoc tests for multiple comparisons (* *p* < 0.05; ** *p* < 0.01; *** *p* < 0.001; **** *p* < 0.0001; n.s., not significant).

## 3. Results

### 3.1. Design and Characterization of mRNA Nanobodies

**Figure 1:**
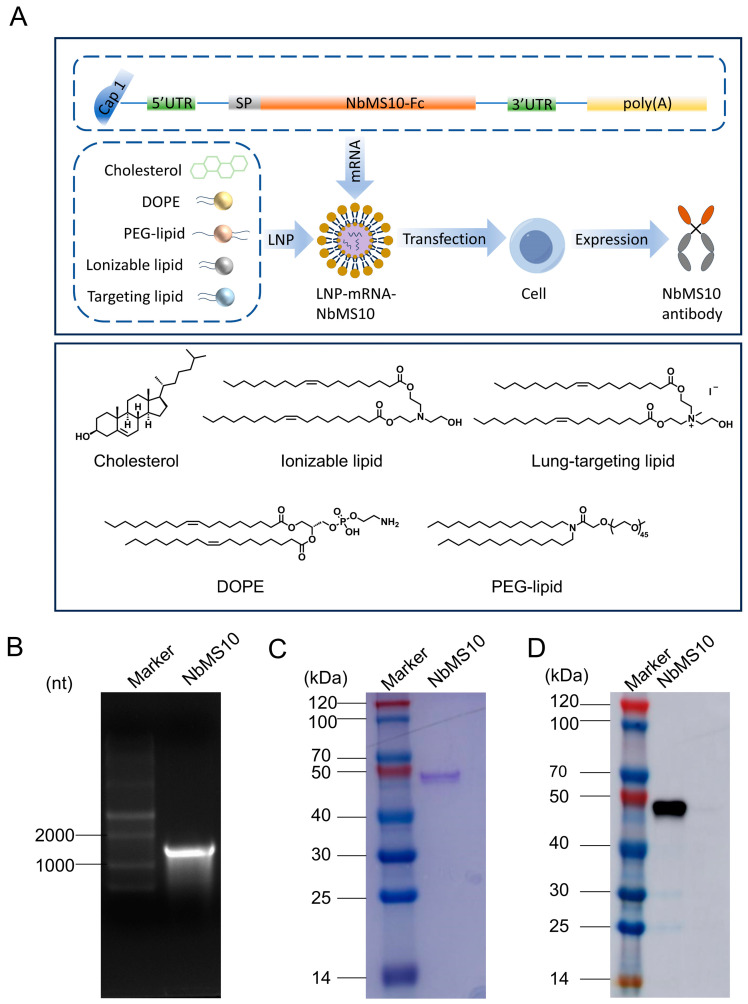
Rational design and characterization of the mRNA antibody. (**A**) Design and encapsulation of the mRNA antibody LNP-mRNA-NbMS10. (**B**) Agarose gel electrophoresis of mRNA samples, visualized under UV light, shows distinct bands corresponding to the expected mRNA size. (**C**) Expression of LNP-mRNA-NbMS10 antibody in the supernatant 24 h post-transfection, analyzed by SDS-PAGE. (**D**) Expression of LNP-mRNA-NbMS10 antibody in the supernatant, confirmed by Western blot analysis using a goat anti-human IgG (H+L) tag antibody.

The NbMS10 sequence was recombinantly constructed using molecular cloning technology from a previously screened nanobody [21]. The tPA signal peptide sequence was first added to the N-terminus of NbMS10, followed by its insertion into the plasmid pFUSE-hIgG1-Fc2, ensuring the presence of a human IgG1 Fc tag at the C-terminus [22]. The recombinant plasmid was initially identified through bacterial liquid PCR and double enzyme digestion, with sequencing confirming the accuracy of all bases. To enhance mRNA translation efficiency, improve stability, reduce immunogenicity, and extend half-life [23], a cap structure and UTR were added to the 5′ end, and a UTR and polyA tail to the 3′ end (Figure 1A). The transcription system was optimized using N1-Me-Pseudo UTP, enabling efficient production of N1-Me-Pseudo UTP-modified RNA via in vitro transcription [24,25]. These modifications reduce the host cell’s immune response. Agarose gel electrophoresis confirmed the integrity of the transcribed mRNA, showing a size of approximately 1300 nt, consistent with the theoretical size (Figure 1B).

Next, mRNA was encapsulated using microfluidic techniques with lung-selective lipid nanoparticles (LNPs), as previously described [19]. LNPs were used to encapsulate mRNA-NbMS10, along with mRNAs encoding two commonly used fluorescent markers, Luciferase and eGFP. The effective diameters, zeta potentials, and encapsulation efficiencies were measured. The results showed that LNP-encapsulated mRNA-NbMS10 had an effective diameter of 95.27 nm, a zeta potential of 4.12 mV, and an encapsulation efficiency of 89%. After encapsulating mRNA-Luci and mRNA-eGFP, the effective diameters increased slightly, the zeta potential decreased, and the encapsulation efficiency remained stable (Table 1). To assess the LNP delivery system’s effectiveness, 293T cells were transfected with LNP-mRNA-NbMS10, and NbMS10 protein in the purified supernatant was analyzed by SDS-PAGE and Western blot (WB). As shown in Figure 1C,D, a band at 45 kDa confirmed successful expression of the NbMS10 protein in cells transfected with LNP-mRNA-NbMS10, verifying the delivery system’s efficacy. The original uncropped images of the Western blot are provided in Figure S1. The integrated density values of the bands in Figure S1 are presented in Table S1.

### 3.2. In Vitro Expression and Protective Efficacy of the mRNA Nanobody

**Figure 2:**
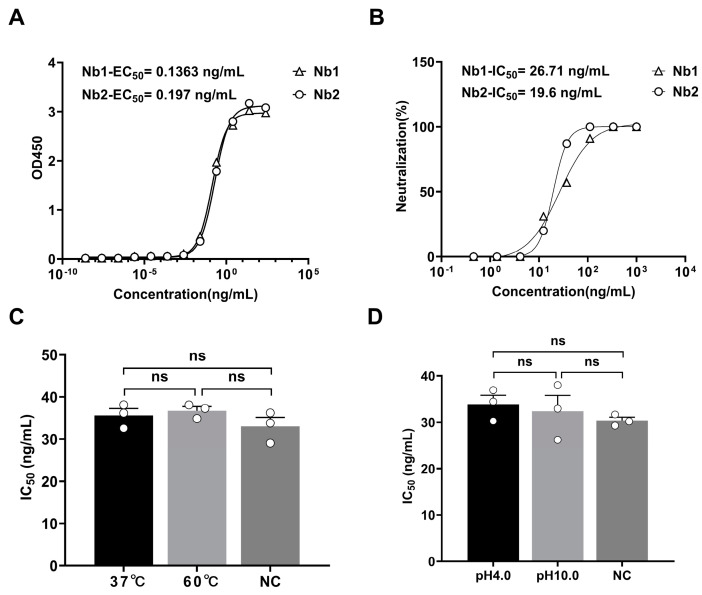
Characterization of the mRNA antibody in cell cultures. (**A**) ELISA curves showing the binding activities of antibodies expressed from DNA and mRNA transfections, with comparable EC_50_ values indicating similar antigen affinities (Nb2−EC_50_ = 0.197 ng/mL, Nb1−EC_50_ = 0.1363 ng/mL). (**B**) Pseudovirus neutralization curves of antibodies expressed from DNA and mRNA transfections, demonstrating similar IC_50_ values (Nb2−IC_50_ = 19.6 ng/mL, Nb1−IC_50_ = 26.71 ng/mL), suggesting equivalent neutralizing potencies. (**C**) Nanobodies were treated at different temperatures (37 °C, 60 °C) for 24 h, followed by measurement of their neutralizing activity against pseudovirus. (**D**) Nanobodies were treated at different pH levels (pH 4.0, 10.0) for 24 h at room temperature, followed by measurement of their neutralizing activity against pseudovirus. (“ns” indicates that the difference between the groups is not statistically significant; *p* > 0.05).

Building on our previous research, the NbMS10 nanobody has shown effective prophylactic and therapeutic potential in protecting a susceptible animal model from a lethal MERS-CoV challenge [21]. In this study, we compared the NbMS10 nanobody expressed via DNA transfection in yeast cells (Nb1) with that expressed through mRNA transfection in 293T cells (Nb2). We evaluated the antigen-binding activity and neutralizing capacity of both nanobodies using enzyme-linked immunosorbent assays (ELISAs) and pseudovirus neutralization assays. The results revealed that both nanobodies had similar EC_50_ values (Nb1-EC_50_ = 0.1363 ng/mL, Nb2-EC_50_ = 0.197 ng/mL) in the ELISA, indicating comparable binding affinities for the target antigen (Figure 2A). Additionally, they exhibited similar IC_50_ values (Nb1-IC_50_ = 26.71 ng/mL, Nb2-IC_50_ = 19.6 ng/mL) in pseudovirus neutralization assays, suggesting equivalent neutralization capabilities against the pseudovirus (Figure 2B) [26]. In conclusion, cells transfected with LNP-mRNA-NbMS10 successfully expressed the NbMS10 nanobody, which demonstrated antigen-binding ability with MERS-CoV S1 and neutralized MERS-CoV pseudoviruses.

Nanobodies are known for their stability under extreme conditions, such as varying pH levels and temperatures. To evaluate this stability, nanobodies were exposed to these harsh conditions and then tested for neutralizing activity against pseudotyped MERS-CoV. After being subjected to two different pH levels (pH 4.0 and 10.0) and thermal stress at 37 °C and 60 °C, the nanobodies retained their neutralizing efficacy against pseudotyped MERS-CoV. The average IC_50_ values were 35.6 at 37 °C, 36.8 at 60 °C, and 33.0 for the negative control (NC). Similarly, at pH 4.0 and 10.0, the average IC_50_ values were 33.9 and 32.4, respectively, compared to 30.4 for the negative control. Statistical analysis revealed no significant differences between the treatment groups, indicating that the nanobodies maintained neutralizing activity similar to their untreated counterparts (Figure 2C,D).

### 3.3. Characterization of the Lung-Selective LNP mRNA Delivery System

**Figure 3:**
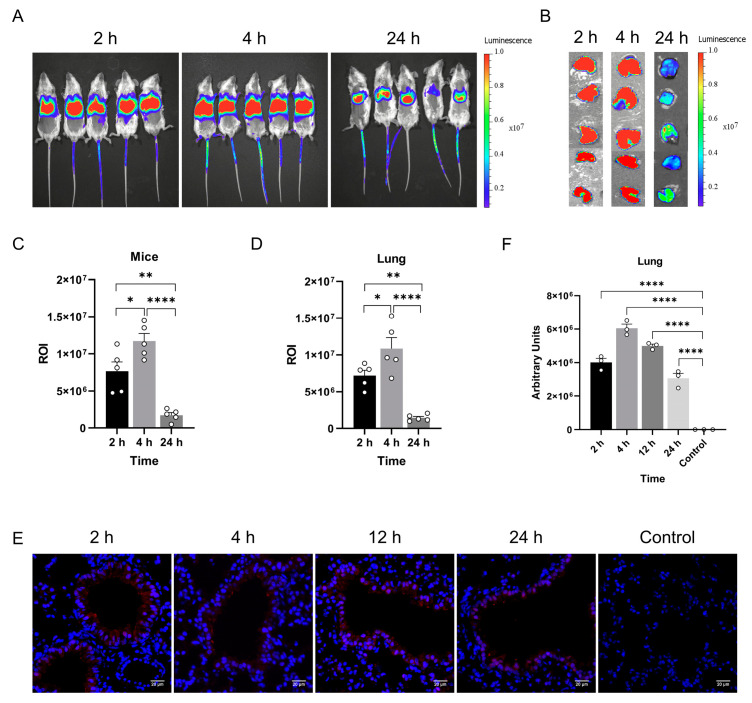
Characterization of lung-targeting lipid nanoparticle systems in live mice. (**A**) In vivo imaging shows the selective accumulation of LNP-mRNA-Luci in the lungs, with imaging results at 2, 4, and 12 h post-intravenous administration (*n* = 5 per group). (**B**) Lung imaging results at 2, 4, and 12 h post-intravenous administration, demonstrating accumulation in the lungs (*n* = 5 per group). (**C**) Statistical analysis of luminescence intensity in mice administered lung-targeting LNP-mRNA-Luci at various time points, with ROI data shown as mean ± SD. (**D**) Statistical analysis of luminescence intensity in lung tissue from mice at various time points following LNP-mRNA-Luci administration, data presented as mean ± SD. (**E**) Confocal images of lung tissue from mice administered lung-targeting LNP-mRNA-eGFP at different time points. Blue represents cell nuclei, and red indicates eGFP expression (*n* = 3 for each time point). (**F**) Confocal images of lung tissue from mice administered LNP-mRNA-eGFP at different time points, with statistical results shown as mean ± SD (*n* = 3). (* *p* < 0.1; ** *p* < 0.01; **** *p* < 0.0001).

We selected previously used positively charged LNPs with an optimized formulation as the delivery vehicle to efficiently transport mRNA to the lungs [19]. The aqueous buffer containing mRNA was mixed with the lipid ethanol solution at a 3:1 (*v/v*) water-to-alcohol ratio, forming mRNA-loaded LNPs. To assess the distribution of this delivery system in mice, in vivo imaging was performed at 2, 4, and 24 h after the intravenous administration of LNP-mRNA-Luci (1 mg/kg). A strong fluorescence signal was detected in the chest region 2 h after injection, persisting for up to 24 h (Figure 3A). After euthanizing the mice, fluorescence signals were specifically observed in the lungs of the mice (Figure 3B). These results confirm that the LNP delivery system effectively targets the lungs.

Living Image 3.0 was used to quantify the fluorescence signal in the region of interest (ROI) from the in vivo imaging. The fluorescence intensity reached 7.66 × 10^6^ at 2 h post-injection, increased to 1.17 × 10^7^ after 4 h, and significantly dropped to 1.70 × 10^7^ at 24 h. Overall, the fluorescence intensity initially increased before declining (Figure 3C). Similarly, the quantification of lung imaging showed a consistent trend, peaking at 4 h and remaining detectable at 24 h (Figure 3D). These results demonstrate that the LNP system effectively targets the lungs and delivers mRNA efficiently.

To identify which cell types in the lungs absorb LNPs and translate mRNA into protein, tissue sections were collected, and eGFP expression was observed using a confocal laser scanning microscope at 2, 4, and 24 h after the intravenous administration of LNP-mRNA-eGFP. The images revealed that high eGFP expression was primarily concentrated in alveolar epithelial cells (Figure 3E). The arbitrary units followed a similar pattern, with an initial rise and subsequent decline (Figure 3F).

### 3.4. Characterization of LNP-mRNA-NbMS10 In Vivo

**Figure 4:**
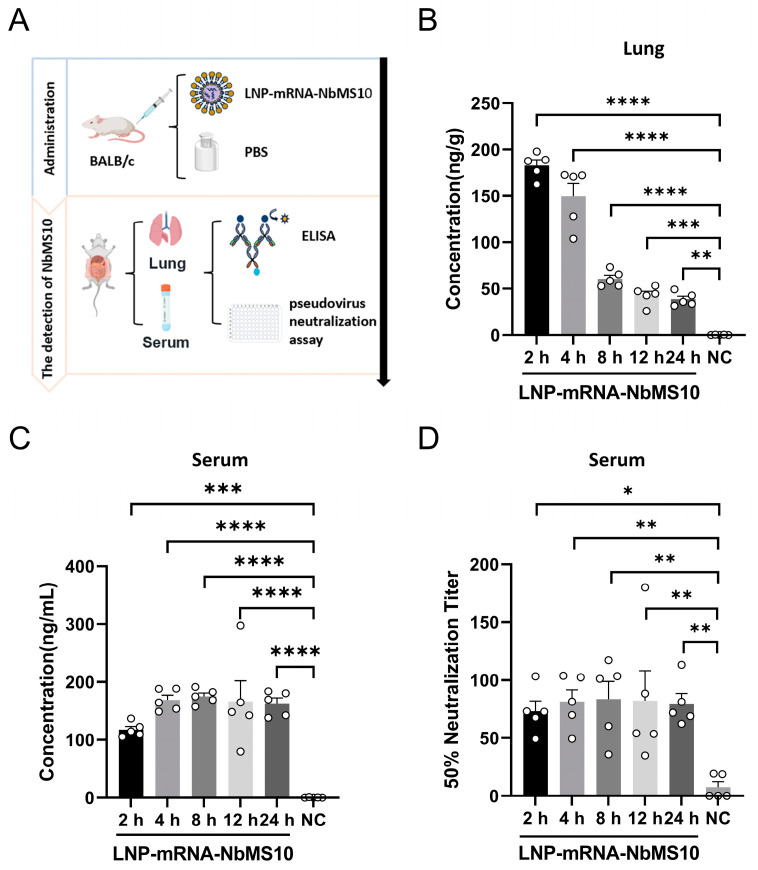
In vivo activity of mRNA-NbMS10. (**A**) Schematic illustrating NbMS10 detection post-injection. (**B**) Lung tissues were collected for NbMS10 detection by ELISA. Female BALB/c mice (*n* = 5 per group) were intravenously administered LNP-mRNA-NbMS10 or PBS. Lung tissue from mice at various time points was measured by ELISA (*n* = 5). Data are shown as mean ± SEM. (**C**) Antibody concentration in serum samples from mice. Female BALB/c mice (*n* = 5 per group) received intravenous injections of LNP-mRNA-NbMS10 or PBS. Serum from various time points was measured by ELISA (*n* = 5 per group). Data are presented as mean ± SEM. (**D**) Antibody NT50 titer of serum samples in mice. Serum was analyzed via a pseudovirus neutralization assay at different time points. Data are shown as mean ± SEM. (* *p* < 0.1; ** *p* < 0.01; *** *p* < 0.001; **** *p* < 0.0001).

LNP-mRNA-NbMS10 was intravenously administered to mice, and lung tissues and serum were collected at various time points (2, 4, 8, 12, and 24 h post-injection) (Figure 4A). Antibody expression was measured using ELISA, with PBS-injected mice serving as the control group. The results showed that the antibody concentration in the lung tissue peaked at 182.7 ng/g 2 h post-injection and gradually decreased over time, but remained significantly higher than the control even after 24 h (Figure 4B). In the serum, LNP-mRNA-NbMS10 expression was detected 2 h post-injection, peaking at 168.8 ng/mL at 4 h. The serum antibody levels stabilized after reaching their peak and persisted for 24 h without a significant decline (Figure 4C).

To evaluate the antiviral efficacy of the NbMS10 nanobody expressed in mice, serum was collected for pseudovirus neutralization assays. Compared to the serum from PBS-injected mice, the serum from LNP-mRNA-NbMS10-injected mice effectively neutralized the pseudovirus. Moreover, the neutralization activity remained robust for 24 h without any declines (Figure 4D).

## 4. Discussion

Current prophylactic and therapeutic strategies for MERS-CoV, including vaccines and monoclonal antibodies, face significant challenges, such as high production costs, limited accessibility, and the need for frequent administration [6]. Additionally, traditional vaccines and antivirals are often ineffective against rapidly mutating viruses and immune escape variants. Recent advances in mRNA technology for treating and preventing infectious diseases have sparked interest in using this platform to produce antibodies [27,28]. The modular nature of mRNA allows for rapid sequence modifications to address emerging strains, offering a versatile and potentially transformative approach to combating a broad range of respiratory pathogens [29,30].

Utilizing mRNA to induce endogenous antibody production overcomes the high cost and complexity associated with traditional protein antibody production. Moreover, mRNA technology simplifies antibody optimization and enables localized antibody expression, an advantage not achievable with conventional methods [31,32,33,34]. Unlike traditional antibodies, which may have limited tissue penetration, nanobodies’ smaller size allows for superior tissue access and antigen targeting [35,36,37,38]. The use of nanobodies in an mRNA context is notably streamlined [39]. Unlike traditional antibody mRNA approaches requiring the transfection of both light and heavy chains, a nanobody can be expressed with a single mRNA transcript, enhancing the efficiency of mRNA utilization [40,41].

In this study, we demonstrated the effectiveness of the lung-targeted LNP-encapsulated mRNA nanobody, LNP-mRNA-NbMS10, in generating serum-neutralizing activity against MERS-CoV. Reducing the immunogenicity of mRNA during design and construction is crucial as many cellular innate immune receptors detect foreign RNA, triggering the release of type I interferons. This leads to the activation of interferon-induced genes and the inhibition of translation [42,43]. To minimize immunogenicity and ensure efficient protein synthesis, we replaced uracil with pseudouridine in our transcripts. Additionally, we incorporated a 5′ cap structure, 5′ and 3′ untranslated regions (UTRs), and a poly(A) tail of 105 nucleotides [38].

After constructing the mRNA encodingthe NbMS10 nanobody, we focused on achieving efficient mRNA delivery. Since the lung is a critical target for preventing infectious pathogens, expressing neutralizing antibodies directly in the lungs offers a promising preventive strategy for respiratory infections. Lung-targeted mRNA delivery provides key advantages over the systemic delivery of recombinant proteins via intravenous injection. First, antibodies translated in the lungs can undergo post-translational modifications, potentially increasing efficacy and stability in vivo. Second, lung-specific mRNA delivery generates a high concentration of neutralizing antibodies at the infection site, essential for preventing or treating respiratory infections. In contrast, traditional delivery methods may not target the mRNA effectively to the desired tissues. Lastly, by delivering mRNA directly to the target tissues, the required mRNA dosage is significantly lower, potentially reducing overall treatment costs compared to protein therapies. Thus, we developed and validated the lung-targeted LNP-mRNA-NbMS10 system for this purpose. By adding the additional permanently cationic targeting lipid and replacing DSPC with 1,2dioleoyl-sn-glycero-3-phosphoethanolamine (DOPE) in the Liver–LNP formulation, we successfully obtained lung-selective LNPs, which were capable of precisely delivering mRNA to the lungs [19].

The results showed that the nanobody NbMS10 expressed in vitro demonstrated strong antigen-binding activity and significant pseudovirus neutralization. Our experimental findings suggest that the antibodies expressed following mRNA transfection have EC_50_ and IC_50_ values comparable to those of the conventional protein antibodies we developed. This similarity highlights the efficacy of mRNA-mediated antibody expression. Furthermore, in vivo studies confirmed that the LNP-mRNA-NbMS10 system specifically targets the lungs, efficiently expresses the NbMS10 nanobody, and maintains prolonged expression. This system’s sustained antiviral activity suggests its potential as a viable alternative to existing MERS-CoV therapies.

Lipid nanoparticles (LNPs), as a key delivery system for mRNA drugs, play a crucial role in protecting mRNA molecules and effectively delivering them to host cells, but their long-term safety remains a concern. Some components of LNPs may be cytotoxic and can elicit different immune responses at various doses [44]. Therefore, although mRNA vaccines have made significant progress in improving the efficacy of COVID-19 vaccines, their safety and potential long-term effects still need to be ensured through ongoing research and monitoring. The ionizable lipid and lung-targeting lipid used in this study contain biodegradable ester bonds to lower the toxicity of these lipids. The hydrophobic tails of these two lipids are oleic acid, whose unsaturated double bond is favorable to alleviate inflammation caused by LNPs. More importantly, our lung-targeting LNPs are expected to cause fewer side effects due to the lung-specific delivery and non-liver accumulation, which are absent in the Pfizer/BioNTech’s Comirnaty COVID-19 mRNA-LNP vaccine. This high lung preference ensures that the lung-targeting LNP can achieve excellent therapy benefits at a low dose in treating lung diseases, while the liver-targeting COVID-19 mRNA-LNP vaccine requires a much higher dose, which probably leads to more severe adverse effects. Additionally, previous studies have demonstrated the biosafety of our LNP by assessing liver and kidney function indicators, hemolysis effects, inflammatory responses, and cytokine levels [19].

Furthermore, mRNA-encoding antibodies are generally considered to be safer than those encoding potentially harmful antigens [45]. This is because antibodies are part of the immune system and typically do not cause disease. When compared to encoding potentially harmful antigens, the use of mRNA-encoding antibodies presents a safer profile, as it entails a lower risk of integration with the host genome and offers enhanced safety for host cells. In short, by encoding antibodies instead of potentially harmful antigens, mRNA provides a safe and effective long-term immunization strategy.

While this study confirmed the effectiveness of the LNP-mRNA-NbMS10 system in preventing and treating MERS infection, exploring combination therapies with other antiviral drugs could further enhance its therapeutic potential. Monotherapy often fails to fully suppress viral replication and transmission, whereas combining drugs with different mechanisms of action may create synergistic effects, targeting multiple stages of the viral life cycle. For instance, the LNP-mRNA-NbMS10 system could be paired with small-molecule viral enzyme inhibitors or host-targeted drugs for a broader antiviral effect. Additionally, co-administration with immunomodulators or anti-inflammatory agents could help mitigate excessive immune responses and reduce tissue damage caused by MERS infection. Optimizing the formulation and dosing of these combination therapies could improve efficacy while minimizing the risk of side effects. Future research should focus on assessing the synergy, safety, and effectiveness of various drug combinations to develop a more comprehensive treatment for MERS. In summary, investigating combination therapies is a promising path for advancing the clinical potential of the LNP-mRNA-NbMS10 system.

## 5. Conclusions

In conclusion, the LNP-mRNA-NbMS10 system marks a significant advancement in the fight against MERS-CoV. Its lung-targeted delivery, stability under diverse conditions, cost-effectiveness, and rapid adaptability make it a promising alternative to existing therapeutic strategies, with the potential to transform the landscape of antiviral therapy.

## Figures and Tables

**Table 1:** Characterization of organ-selective lipid nanoparticles.

mRNA	Eff.Diam. (nm)	Zeta Potential	Encapsulation Rate (%)
NbMS10	95.27	4.12	89
Luciferase	101.51	2.79	92
eGFP	106.73	2.50	91

## Data Availability

The data that support the findings of this study are available from the corresponding author upon reasonable request.

## Supplementary Materials

The following supporting information can be downloaded at https://www.mdpi.com/article/10.3390/vaccines12121315/s1, Figure S1: In vitro expression validation of LNP-mRNA-NbMS10; Table S1: Source data for Figure S1.
