# Supplementary material for: Lung-Selective Delivery of mRNA-Encoding Anti-MERS-CoV Nanobody Exhibits Neutralizing Activity Both In Vitro and In Vivo
Source: Vaccines (Basel). 2024 Nov 24;12(12):1315. doi: 10.3390/vaccines12121315 (PMC11680347; doi:10.3390/vaccines12121315)
Supplement: Supplementary file 1 [file vaccines-12-01315-s001.zip › vaccines-3276369-supplementary.pdf]

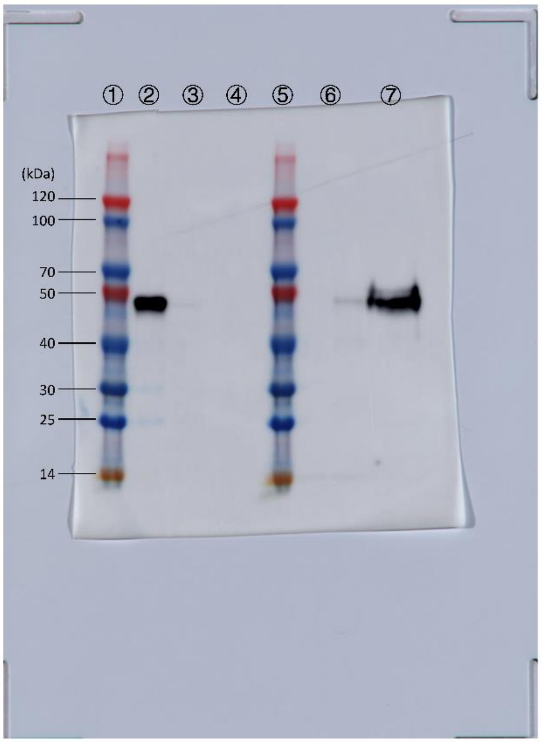

Figure S1. In vitro expression validation of LNP-mRNA-NbMS10. Expression of LNP-mRNA-NbMS10 antibody in the supernatant, confirmed by western blot analysis using a goat anti-human IgG (H+L) tag antibody (①, ⑤: Marker; ②: NbMS10-Fc ; ③, ④, ⑥, ⑦: Not related.).

Table S1. Source data for Figure S1.

Target	Integrated Density	Relative Expression
NbMS10	370030	1.50
50 kDa	246278	
